# Advancements and prospects of novel biologicals for myasthenia gravis: toward personalized treatment based on autoantibody specificities

**DOI:** 10.3389/fphar.2024.1370411

**Published:** 2024-05-27

**Authors:** Chi Ma, Dan Liu, Benqiao Wang, Yingying Yang, Ruixia Zhu

**Affiliations:** Department of Neurology, The First Affiliated Hospital of China Medical University, Shenyang, China

**Keywords:** myasthenia gravis, acetylcholine receptor myasthenia gravis, muscle-specific tyrosine kinase myasthenia gravis, personalized treatment, immunotherapy

## Abstract

Myasthenia gravis (MG) is an antibody-mediated autoimmune disease with a prevalence of 150–250 cases per million individuals. Autoantibodies include long-lived antibodies against the acetylcholine receptor (AChR), mainly of the IgG1 subclass, and IgG4, produced almost exclusively by short-lived plasmablasts, which are prevalent in muscle-specific tyrosine kinase (MuSK) myasthenia gravis. Numerous investigations have demonstrated that MG patients receiving conventional medication today still do not possess satisfactory symptom control, indicating a substantial disease burden. Subsequently, based on the type of the autoantibody and the pathogenesis, we synthesized the published material to date and reached a conclusion regarding the literature related to personalized targeted therapy for MG. Novel agents for AChR MG have shown their efficacy in clinical research, such as complement inhibitors, FcRn receptor antagonists, and B-cell activating factor (BAFF) inhibitors. Rituximab, a representative drug of anti-CD20 therapy, has demonstrated benefits in treatment of MuSK MG patients. Due to the existence of low-affinity antibodies or unidentified antibodies that are inaccessible by existing methods, the treatment for seronegative MG remains complicated; thus, special testing and therapy considerations are necessary. It may be advantageous to initiate the application of novel biologicals at an early stage of the disease. Currently, therapies can also be combined and individualized according to different types of antibodies. With such a wide range of drugs, how to tailor treatment strategies to patients with various conditions and find the most suitable solution for each MG profile are our necessary and urgent aims.

## 1 Introduction

Myasthenia gravis (MG) is a rare autoimmune disorder with local or generalized muscle weakness as the main symptom and is caused by neuromuscular junction (NMJ) transmission defects (Gilhus, 2016; Gilhus et al., 2019). The primary clinical symptom of MG patients is the fluctuating muscle weakness of the ocular, limb, and axial muscles that worsens after exercise, which has an enormous impact on their health-related quality of life (HR-QOL). Consequently, muscle function typically peaks in the morning and gradually aggravates throughout the day. The course of this disease can be long term, with a tendency to relapse, and can even be life-threatening when bulbar and respiratory muscles are involved, so it is necessary to find the root cause of MG and develop specific treatments. Binding of pathogenic immunoglobulin IgG and functionally important components to the postsynaptic membrane is an important part of the MG mechanism (Gilhus et al., 2019). Therefore, the development of antibody treatment is important to improve diagnostic processes and develop individualized medical strategies. According to international consensus guidelines, the aim of MG treatment is to achieve minimum manifestation state (MMS) or better (in MMS, MG has no symptoms or dysfunction, but there is some weakness on certain muscle tests) with no more than grade 1 adverse effects (Sanders et al., 2016).

**TABLE 1:** Summary of the main clinical trials of novel targeted drugs in myasthenia gravis (MG) subgroup patients.

Author	Year	MG subgroups	Patient	Therapy	Study design	Outcome
Howard JF et al.	2017	AChR-positive gMG Inclusion criteria:	125	Eculizumab	Randomized, double-blind, placebo-controlled, multi-center, phase III study (REGAIN)	MG-ADL score −4.2 vs. −2.3 points (*p* = 0.0058)
		MG-ADL score of 6 or more				QMG score −4.6 vs. −1.6 points (*p* = 0.0006)
		MGFA classes II–IV				MG-QoL score −12.6 vs. −5.4 points (*p* = 0.0010)
Anderson et al.	2020	Patients who completed the REGAIN study	125	Eculizumab	Open-label extension study	Neuro-QoL fatigue scores are correlated with QMG, MG-ADL, and MG-QoL scores
Muppidi et al.	2019	Patients who completed the REGAIN study	117	Eculizumab	Interim analysis of safety and efficacy	Sustained treatment effect for 3 years
Howard JF et al.	2022	Adults with AChR-positive gMG	175	Rozanolixizumab	Randomized, double-blind, placebo-controlled, phase III study (CHAMPION)	MG-ADL score −3.1 vs. −1.4 points (*p* < 0.001)
		MG-ADL score of 6 or more				QMG score −2.8 vs. −0.8 points (*p* < 0.001)
		MGFA classes II–IV				MG-QoL score 15 −3.3 vs. −1.6 points (*p* = NA)
Howard JF et al.	2023	Adults with AChR-positive gMG	174	Zilucoplan	Randomized, double-blind, placebo-controlled, phase III study (RAISE)	MG-ADL score change: least squares mean change −4.39 [95% CI: –5.28 to −3.50] vs. −2.30 [–3.17 to −1.43]
		MG-ADL score of 6 or more				
		MGFA classes II–IV				
Howard JF et al.	2019	AChR-positive gMG Inclusion criteria: MG-ADL score 5 or higher	24	Efgartigimod	Randomized, double-blind, placebo-controlled, 15-center, phase II study	Significant changes (75%) in QMG, MG-QoL, MG-ADL, and MG composite disease severity scores
		MGFA classes II–IVa				
Howard JF et al.	2021	gMG inclusion criteria:	167	Efgartigimod	Randomized, double-blind, placebo-controlled, phase III study (ADAPT)	MG-ADL score −4.45 vs. −1.84 points (*p* < 0.001)
		MG-ADL score ≥5				QMG score −6.21 vs. −1.01 points (*p* < 0.001)
		Stable dose of ≥1 treatment of gMG				MG-QoL score −7.7 vs. −2.61 points (*p* < 0.05)
		MGFA classes II–IV				
Bril V et al.	2023	AChR or MuSK antibody-positive gMG	200	Rozanolixizumab	Randomized, double-blind, placebo-controlled, phase III study (MycarinG)	7 mg/kg,10 mg/kg group vs. placebo group
		MG-ADL score of 3 or more				MG-ADL score −3.37,-3.40 vs. −0.78 points (*p* < 0.0001)
		MGFA classes II–IVa				QMG score −5.40,-6.67 vs. −1.92 points (*p* < 0.0001)
		QMG score of at least 11				
Remegen Co., Ltd.	2022	AChR-positive gMG Inclusion criteria:	29	Telitacicept	Randomized, multi-center, open-label, phase II study	Average score decreased by 7.7 in the 160-mg group QMG and 9.6 in the 240-mg group
		accept standard treatment				
Hehir et al.	2017	MuSK-positive MG	55	Rituximab	Blinded, multi-center, prospective review	58% patients had an MGSTI level 2 or better
						67% patients have an MGFA PIS score of MM or better
Topakian et al.	2019	MG with rituximab treatment	56	Rituximab	Retrospective nationwide study	Remission: MuSK MG vs. AChR MG (71.4% vs. 35.9%, *p* = 0.022)
Brauner et al.	2020	Non-MuSK gMG	72	Rituximab	Retrospective, cohort study	Median time to remission: new onset 7 < refractory 16 months
		Rituximab treatment, new onset or refractory				Rituximab 7 < conventional therapies for 11 months
Li H et al.	2021	New-onset gMG	19	Rituximab	Retrospective case series study	Take rituximab within 3 months from onset, 89% patients were relapse-free
						Significant decrease in MG-ADL, QMG, MG-QoL score
Nowak et al.	2021	AChR-positive gMG Inclusion criteria: MGFA classes II–IV prednisone ≥15 mg/day	52	Rituximab	Randomized, double-blind, placebo-controlled, multicenter phase II study (Beat MG)	MG-ADL score −2.7 vs. −2.0 points (*p* = 0.73)
						QMG score −4.0 vs. −1.7 points (*p* = 0.39)
						MG-QoL score −8.0 vs.7.5 points (*p* = 0.70)
Piehl F et al.	2022	New-onset MG patients with a QMG score of 6 or more	47	Rituximab	Randomized, double-blind, placebo-controlled study (RINOMAX)	MG-ADL score −1.7 vs. −0.5 points (*p* = 0.34)
						QMG score −6.9 vs. −5.8 points (*p* = 0.79)
						MG-QoL score −9.2 vs.7.0 points (*p* = 0.47)
Du et al.	2022	New-onset AChR MG	13	Rituximab	Prospective single arm study	All patients achieved MM or better with a low dose of rituximab over 19 months

*gMG*, generalized myasthenia gravis; *MG-ADL*, Myasthenia Gravis-Activities of Daily Living; *MGFA*, Myasthenia Gravis foundation of America; *NSIST*, non-steroidal immunosuppressive therapy; *QMG*, quantitative myasthenia gravis; *MG-QoL*, myasthenia gravis quality of life; *MGSTI*, myasthenia gravis status and treatment intensity, *MGFA PIS*, Myasthenia gravis Foundation of America Post-intervention Status.

**TABLE 2:** Response of AChR ab-positive MG and MuSK ab-positive MG to different treatment options.

Treatment	AChR ab-positive MG	MuSK ab-positive MG
Thymectomy	Benefit	No observed benefit
IVIG	Benefit	No observed benefit
Anti-CD20	Partial benefit	Clinical benefit
Complement inhibitor	Benefit	No observed benefit
FcRn antagonist	Benefit	Benefit to be investigated
Telitacicept	Benefit	Benefit
BAFF inhibitor	Benefit	Benefit
IL-6R inhibitor	Uncertainty of benefit	Uncertainty of benefit

The overall prevalence of MG is 150–250 cases per million people, with an estimated annual incidence of 8–10 cases/million person-years (Gilhus et al., 2019). The prevalence and incidence of MG varies among different subgroups. According to different antigen targets, it can be divided into subgroups such as acetylcholine receptor (AChR) antibodies, muscle-specific tyrosine kinase (MuSK) antibodies, and lipoprotein receptor-related protein 4 (LRP4) antibodies. Nicotinic acetylcholine receptor antibodies, which can be found in approximately 80% of MG patients, are the most common autoantibodies (Gilhus, 2016). The remaining approximately 15% are antibodies against MuSK and LRP4, and a small proportion has not yet been detected, referred to as seronegative myasthenia gravis (SNMG) antibodies (Fichtner et al., 2020).

Although the clinical presentation of the different subgroups is similar, there are significant differences in their nature and origin. Short-lived and long-lived antibody-secreting cells can be distinguished from B cells, which are routed through two pathways: the extrafollicular pathway and the germinal center (GC) pathway. The extrafollicular pathway, in which B cells develop into short-lived plasmablasts (SLPBs) at extrafollicular sites, is the first stage of the humoral immune response. The initial stage of the T-cell antigen-dependent immune response is in the T-cell zone, where naive B cells develop and differentiate with the help of cytokines secreted by T cells. SLPBs and GC-independent memory B cells are eventually generated as a result of activation of B-cell receptors (BCRs) by antigens on follicular dendritic cells (FDCs), which are subsequently presented to T follicular helper (Tfh) cells via major histocompatibility complex-II (MHC-II). In the next phase, B cells undergo somatic hypermutation in the dark zone of the GC; then circulate between light and dark zones, being selected by the light zone by the affinity of the BCR for antigens; and finally differentiate into memory B cells and long-lived plasma cells (LLPCs) with the assistance of Tfh cells, the latter leaving the GC and usually accumulating in a survival niche, such as the bone marrow. Stimulated by autoantibodies and assisted by T cells, B cells are further activated and differentiated into LLPCs and SLPBs, which, in turn, produce different autoantibodies. The data showed that in AChR MG, which was dominated by IgG1, the production of autoantibodies was more related to antigen-specific LLPCs, while in MuSK MG (the main antibody is IgG4), the role of SLPBs has been demonstrated (Zografou et al., 2021).

Currently, therapies can be combined and individualized according to different types of antibodies. With the goal of restoring muscle strength and reducing symptoms as much as possible, many drugs and treatment strategies have emerged from different facets nowadays. Cholinesterase inhibitors provide temporary relief and symptomatic improvement by increasing the bioavailability of acetylcholine at the neuromuscular junctions (Conti-Fine et al., 2006; Gilhus et al., 2019). Immunotherapy includes the use of corticosteroids, as well as immunosuppressants such as antimetabolites azathioprine and mycophenolate mofetil to inhibit B cells and T cells, T and NK cell inhibitors such as cyclosporine and tacrolimus, and plasma exchange (PLEX) and intravenous immunoglobulin (IVIG) as means of rapid-acting agents that are important in the acute exacerbation of MG. In addition, 10%–15% of MG patients develop complications such as thymoma, and thymectomy is feasible if the patient’s condition is stable. For patients without thymoma, if the patient is AChR antibody-positive and has early-onset disease, thymectomy is also available as a treatment to improve symptoms. For anti-MuSK antibody-positive MG patients, cholinesterase inhibitors are unlikely to work and may even cause adverse effects, but they respond well to B-cell inhibitors, especially rituximab. There are also a number of complement inhibitors, FcRn antagonists, and other emerging drugs that are already on the market or under evaluation (Gilhus, 2016). These emerging biologics offer new hope to suffering patients, although their widespread use is still a long way off (Table 1). An evaluation of the Myasthenia Gravis Patient Registry (MGR) concluded that many patients still suffer from adverse effects, that only a small percentage of them receive treatments other than traditional immunotherapies, i.e., about 12% (Cutter et al., 2019), and that few patients reached minimal symptom expression (MSE) after 1 year of treatment (Lee et al., 2022). Data from not only Sweden but also five European countries suggest that the majority of patients still do not have favorable symptom control, indicating a high disease burden (Petersson et al., 2021; Mahic et al., 2023). Patient-acceptable symptom state (PASS) is a new concept to anchor thresholds for MG rating scales derived from the patients’ views; it may bring new opinions to the evaluation of future treatment trials (Mendoza et al., 2020). All of this shows that effective treatments for MG are still a long way off and that much remains to be done to meet the real needs of patients. Narrowing the gap in our understanding of the condition between patients and physicians may lead to better outcomes.

Relying on conventional drugs alone has proven to be very inadequate at this stage, and the emergence of targeted drugs in recent years has increased the options for treating MG. A popular topic in the treatment of MG is how to logically develop tailored treatment regimens for patients based on various antibody types, taking into account the efficacy, safety, and accessibility. In this article, we review the emerging targeted drugs and, in light of the pathogenic mechanisms of MG, propose the corresponding therapeutic regimens that provide a basis for drug selection.

## 2 AChR-MG

### 2.1 Clinical features of AChR-MG

In most AChR antibody-positive patients with MG, there is a bimodal pattern of age at onset, with two peaks in the thirties and between 70 and 80 years of age. Early-onset MG refers to age at onset less than 50 years, predominantly women, whereas late-onset MG refers to age at onset greater than 50 years, with a higher incidence in men (Yi et al., 2018). Thymic hyperplasia characterizes early-onset MG, whereas thymic atrophy represents late-onset MG. B cells of thymic hyperplasia are involved in the production of AChR antibodies. Elderly patients are prone to exacerbation and even life-threatening disease due to comorbidities. The first manifestation of MG is usually eye symptoms such as ptosis, double vision, or both, and there are no symptoms of weakness in other parts of the body (Gilhus, 2016). Autoantibodies could not be detected in 40%–50% of ocular MG cases. However, this does not rule out the possibility that their existence has a causal relationship. In the early stages of the disease, autoantibody titers may be lower than the detection level of conventional methods and/or may be accumulated at NMJs, making them undetectable in serum (Fichtner et al., 2020).

### 2.2 Mechanism of AChR antibody production

AChR antibodies, as mainly consisted by the IgG1 category, are closely related to LLPCs. B cells differentiate into SLPBs, the molecule that generates IgG4, as discussed below. This process is phase 1 of the primary response. In the subsequent phase 2, candidate B cells are selected by the GC light zone based on the affinity of the BCR to antigens. The selected GC B cells encounter antigens on FDCs and are then presented to Tfh cells to promote the interaction with antigens. Thereafter, the activated GC B cells turn into LLPCs that leave the GC and undergo homing to viable niches, such as bone marrow or thymus. Compared with peripheral blood, thymus, and lymph nodes, the concentration of autoantibodies in cultured bone marrow cells of AChR MG patients is higher, demonstrating the involvement of LLPCs in the production of AChR autoantibodies (Akkaya et al., 2020; Zografou et al., 2021).

### 2.3 Pathogenesis of AChR-MG

**FIGURE 1:**
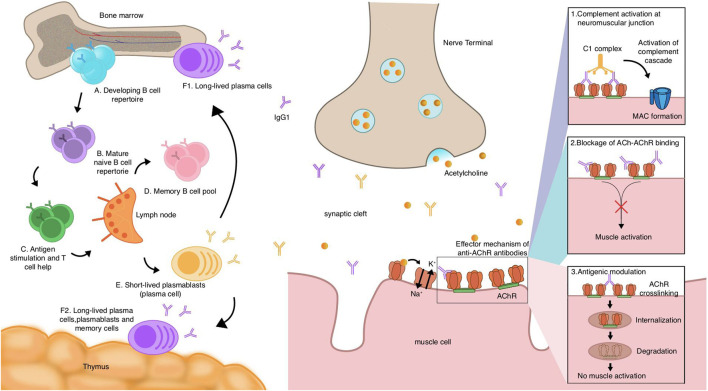
Mechanistic hypothesis of acetylcholine receptor (AChR) myasthenia gravis (MG) autoantibody production and pathogenesis of AChR antibodies.

In MG with AChR antibodies, most of the antibodies belong to the IgG1 subtype (Yi et al., 2018). Acetylcholine receptor is a transmembrane protein consisting of five subunits, i.e., two identical α subunits, β, γ (mostly present only in the embryonic stage), ε (in adults), and δ subunits (Conti-Fine et al., 2006). The α subunit is an important structural element in the function of the AChR because the main immunofunctional region (MIR) on the α subunit is the site where acetylcholine binds to the AChR (Gilhus et al., 2019). The pathogenic mechanisms of AChR antibodies can be divided into three main aspects. First is complement activation, which is also an effector function of immunoglobulin. The binding of the complement to the antibody triggers the activation of the complement cascade, leading to the formation of the membrane attack complex (MAC) and secondary destruction of the muscle membrane of the NMJ via the reduction in postsynaptic membrane folds (Conti-Fine et al., 2006; Gilhus et al., 2019). The complement plays a pivotal role in the pathophysiological mechanism of MG, so the use of complement inhibitors has resulted in successful therapeutic outcomes (Muppidi et al., 2019). In addition, the process of damage induced by complement activation depends not only on the complement itself but also on the properties of the NMJ. For example, the NMJs of extrinsic ocular muscles (EOMs) express less intrinsic complement regulators than normal, making them more susceptible to complement-mediated postsynaptic membrane injury (Kaminski et al., 2004). The second important pathogenic mechanism is antigen modulation, which is the ability of an antibody to bind to two antigen molecules (Fichtner et al., 2020). The two binding sites of the autoantibodies cross-link two acetylcholine receptors by bivalent bonding, leading to the acceleration of internalization and degradation of the AChR. When the degree of endocytosis exceeds the compensatory range, there are fewer AChRs on the postsynaptic membrane, and symptoms of myasthenia occur. Although all Igs have two binding sites, differences in epitope location also affect the ability of antibodies to cross-link another AChR, so not all antibodies have the function of cross-linking with the receptors to induce their internalization and eventual degradation (Conti-Fine et al., 2006). The third, less common, is a functional blockade of ACh–AChR binding. ACh binding is inhibited by antibodies once they bind to or near the acetylcholine binding site on the AChR, resulting in disruption of signaling. Notably, Abs that bind to the α subunit are more pathogenic than other abs, and disease severity is also correlated with Ab epitope patterns (Gilhus et al., 2019). It is of interest to assess disease severity by monitoring AChR α subunits. According to reports, AChR antibodies can be categorized into binding, blocking, and modulating antibodies according to their heterogeneous nature. As it has been shown that more severe generalized MG and MG crises can occur in patients with both binding and blocking antibodies, the detection of both antibodies is helpful in predicting the prognosis. In this regard, the application of a set of quantifying the autoantibodies that mediate either binding or blocking, even modulating, may be better related to disease severity than simply measuring AChR-binding titers (Kang et al., 2015). The heterogeneity of antibodies can also influence the effect of drugs specific for different targets (Figure 1).

The pathway of AChR MG antibody production starts with naive B cells. After development and maturity (A,B), relying on the assistance of T cells, they encounter autoantigens and get stimulated in the lymph nodes (C). They can then differentiate into memory B cells (D), short-lived plasmablasts (E), and long-lived plasma cells (F1,F2), which the thymuses or bone marrows of some AChR MG patients include. Plasma cells and plasmablasts may contribute to the production of autoantibodies, most of which belong to the IgG1 subclass, and it is thought that long-lived plasma cells are dominant in the production of AChR autoantibodies. AChR binding to ACh causes the opening of channels to allow sodium ions in and potassium ions out, which, in turn, causes action potentials of muscle cells. Autoantibodies of the AChR exert their effects through three mechanisms: 1) the complement is activated by antibodies bound to the AChR, triggering a cascade reaction that forms the MAC, causing tissue destruction and inflammation; 2) autoantibodies directly block the binding sites of the AChR, and the effects of the AChR are then inhibited. Following this, the action of ACh is inhibited, and thereby, the muscle cannot be activated; and 3) antigenic modulation. Autoantibodies cross-link two AChRs, leading to the internalization and eventual degradation of the AChR, a process that reduces the amount of AChRs on the postsynaptic membrane.

### 2.4 Therapeutic strategies for AChR-MG

Current conventional treatments include pyridostigmine and various immunosuppressants such as the calcineurin inhibitor tacrolimus and the DNA disruptor azathioprine, all of which are effective in treating AChR MG. Like other immune disorders, AChR MG benefits from IVIG; plasmapheresis and IVIG are taken as options to rapidly control the condition in an acute attack of MG, and they are also used before steroid hormone therapy or thymectomy to prevent exacerbation. Furthermore, thymectomy can be beneficial in patients with early-onset AChR MG or with thymoma (Gilhus et al., 2019). Although conventional immunotherapies achieve most remissions, continued exposure to immunosuppressants in order to maintain long-term steady state increases the likelihood of infection, tumor, and organ dysfunction due to the non-specificity of the immune targets of the drugs. Recently, the advancement of a number of emerging targeted biologic agents has provided new options for future MG therapy, and MG treatment is entering a new era of effective targeted immunotherapies. However, there is still an unmet need for treatment, and there are still some patients who have not achieved MMS, which illustrates the dilemma of MG treatment.

#### 2.4.1 The partial efficacy of anti-CD20 therapy

CD20 is a surface protein found on almost all B cells with the exception of pro B, pre B, plasmablasts, and plasma cells (Fichtner et al., 2020). The anti-CD20 monoclonal antibody drug rituximab works in MG therapy by depleting CD20^+^ B cells and diminishing the production of pathogenic antibodies. Based on the fact that post-rituximab antibody titers reflect the therapeutic effect, a significant decrease in response after rituximab induction may reflect the short-lived nature of antibody-secreting cells (Zografou et al., 2021).

Several studies have been conducted in recent years to investigate the use of rituximab in non-MuSK MG, suggesting a partial benefit in AChR MG. A retrospective cohort study conducted by Brauner et al. (2020) to explore the effect of rituximab in new-onset (defined as MG not exposed to immunosuppressive agents other than glucocorticoids and with an onset of less than 12 months) *versus* refractory generalized MG affirmed the role of rituximab and revealed that its early application to new-onset MG is a more favorable and effective option. Patients receiving rituximab early on had a lower rate of needing rescue treatment, but the evidence is limited by the fact that this trial was retrospective and not randomized. Piehl et al. (2022) conducted a randomized, double-blind clinical trial, which, to some extent, compensates for the limitation of retrospective studies to a certain degree. The conclusion was that low-dose (500 mg) rituximab led to a higher probability of new-onset MG patients achieving minimum MG performance and a lower likelihood of rescue medicines than in placebo. Further research has been conducted in this direction, refined to the exploration of individualized regimens. Li H. et al. (2021) and Du et al. (2022) showed that the early use of low-dose rituximab provides sustained clinical improvement in new-onset MG and that individualized rituximab dosing regimens achieve better clinical improvement. In addition, the early use of rituximab may minimize steroid doses in long-term follow-ups (Du et al., 2022). Repeated applications of low-dose rituximab are also effective in refractory MG, although early applications were more beneficial (Li T. et al., 2021). However, Nowak et al. (2021) conducted a phase II trial of rituximab in AChR Ab-positive gMG (general MG), in which corticosteroid reduction, which was the primary endpoint, did not show efficacy compared to the placebo, and the meaningful steroid-sparing effect is of low probability. The selection of patients with mild baseline symptoms and the inappropriate setting of the primary outcome may have contributed to the lack of positive results in this trial. The latest international consensus has taken rituximab only as an option when patients with AChR MG cannot tolerate other immunosuppressants (Narayanaswami et al., 2021). Repeated administration of rituximab enhances B-cell depletion, preventing the generation of new LLPCs, while allowing a slow decrease in existing LLPCs (Zografou et al., 2021), while B-cell depletion was positively correlated with symptom improvement (Anderson et al., 2016), which implies that rituximab also plays a certain role in the treatment of AChR MG. However, given the deficiency of CD20 on the surface of long-lived plasma cells, AChR MG is partially treated using CD20 drugs. MuSK MG had a better response to anti-CD20 therapy than AChR MG, including earlier remissions and fewer hospitalizations (Litchman et al., 2020). Although autoantibody concentrations were also reduced, some showed very slight decreases, while others were recalcitrant or less pronounced. The above results suggest that not only are LLPCs present in patients with large amounts of IgG1 but SLPBs also exist (Zografou et al., 2021). Thus, the use of rituximab in AChR MG patients still requires further randomized controlled trials with large sample sizes, as well as an assessment of the long-term benefit–risk balance of rituximab in new-onset gMG.

#### 2.4.2 The efficacy of complement inhibitors

Eculizumab is a recombinant humanized monoclonal antibody that reduces postsynaptic membrane damage by inhibiting the activation of the terminal complement (Fichtner et al., 2020). Specifically, eculizumab binds C5 protein with high affinity and blocks C5 convertase from binding and subsequent cleavage into pro-inflammatory C5a (a potential anaphylatoxin) and C5b (a fundamental component of MAC formation), thereby reducing the effect of the MAC on NMJ damage. The complement plays a vital role in the pathophysiological mechanisms of autoantibody pathogenesis, especially in AChR Ab-positive patients. Eculizumab, the first approved effective targeted complement drug, is indicated for gMG in the United States and refractory MG in Europe, as well as for AChR ab-positive gMG patients in Japan, whose symptoms are not improved by PLEX or IVIG (Dhillon, 2018). In 2023, eculizumab was approved by the National Medical Products Administration of China for AChR refractory gMG. The efficacy of the drug was measured by a multicenter, randomized, double-blind, placebo-controlled trial to assess the safety and efficacy of eculizumab in patients with refractory gMG in a study conducted by Howard et al. (2017); there was no statistically significant advantage of eculizumab over the placebo group, which was based on the results of phase III of the study. Although the primary endpoint (change from baseline in the Myasthenia Gravis-Activities of Daily Living [MG-ADL] scale, as assessed by worst-bank ANCOVA) was not reached, analysis of the *post hoc* sensitivity of this outcome compared with the placebo group supported the ameliorative effect of eculizumab on symptoms; in addition, the HR-QOL measures and muscle strength (QMG score) in the secondary analysis of the REGAIN study showed significant improvement. The extension study of REGAIN also noted that the therapeutic effects of eculizumab were sustained over at least 52 weeks of continuous treatment. The heterogeneity of the AChR antibody was also reflected in the trial, with 40% of patients failing to meet the primary endpoint and a wide variability in the degree of response to eculizumab among patients, both suggesting heterogeneity among patients in the relative proportions of complement activation, blocking, and modulating functions mediated by autoantibodies. This confirms the need for more in-depth antibody-specific studies and targeted drug development. The adverse effects of eculizumab are few and mild to moderate, the most common being headache. The risk of *Neisseria meningitidis* infection increased with eculizumab treatment, requiring vaccination against *Neisseria meningitidis* before taking eculizumab. Despite the high acquisition of cost and poor response to the drug due to C5 gene variants, eculizumab is still a valuable emerging drug (Nishimura et al., 2014; Cai et al., 2019). According to international consensus, when treating severe, refractory, AChR Ab-positive gMG, eculizumab should be taken into consideration (median 9, range 2–9), which may be very susceptible to MG crisis (Sanders et al., 2016).

Ravulizumab, a C5 complement inhibitor designed on the basis of eculizumab with an extended half-life of elimination and duration of action, is an upgraded version of eculizumab with long-lasting effects, which may reduce the burden of administration (Vu et al., 2023). The CHAMPION MG phase III trial showed that ravulizumab is well-suited to provide long-term therapeutic effects with few side effects in adults with AChR Ab-positive gMG, and more applications in the future will provide insights (Vu et al., 2022).

A macrocyclic peptide called zilucoplan prevents the cleavage of C5 into C5a and C5b, thereby blocking the cascade reaction. Phase III trials evaluating its safety and efficacy in patients with AChR gMG showed a clinically significant reduction in MG-ADL scores compared to the placebo group and a rapid onset of action. No severe adverse effects were observed (Dalakas, 2020; Howard et al., 2023). Given that the drug has been approved by the United States FDA for use in patients with AChR-positive generalized MG, the availability of future data on a larger scale with longer follow-ups is expected.

Ocular MG has higher MG susceptibility because the NMJ of its EOM lacks intrinsic complement regulators. Complement inhibitors can be considered a treatment intervention for ocular MG because complement regulators shield the NMJ from complement-mediated tissue damage (Kaminski et al., 2004).

#### 2.4.3 The efficacy of neonatal Fc receptor antagonists

Neonatal Fc receptor (FcRn) is a molecule found mainly in the reticuloendothelial system that recycles IgG and regulates its transport (Borghi et al., 2020). Binding of IgG antibodies to FcRn enables Abs to avoid lysosomal degradation, extending the half-life, which is approximately four times that of other Ig antibodies without FcRn protection, and involves in IgG homeostasis (Roopenian and Akilesh, 2007; Lünemann, 2021). In addition to IgG synthesis, recycling and circulation play a pivotal role in the 3–4-week-long half-life of IgG and high concentration in serum. The development of drugs such as FcRn antagonists is based on this process, which promotes the reduction in circulating IgG by competitively blocking the binding of Ab to FcRn with high affinity for FcRn, thereby achieving the effect of alleviating MG symptoms (Fichtner et al., 2020).

A humanized IgG1 Fc fragment called efgartigimod (ARGX-113) specifically blocks the recirculation of IgG to accelerate the reduction in serum concentrations of AChR autoantibodies (mainly IgG1 and IgG3) (Howard et al., 2021). From the conclusions reached by Howard et al. (2019) in the exploratory phase II trial, it can be understood that efgartigimod caused a rapid and significant decrease in IgG levels, producing a significant separation from the placebo group 1 week after the initial injection, although the placebo exerted a significant effect in other trials as well. Efgartigimod showed good efficacy and tolerability in patients administered the drug, with no serious adverse effects reported and no significant adverse consequences in combination with other drugs. Using four scales, MG-ADL, QMG, Myasthenia Gravis Composite Disease Severity Scores, and the revised 15-item Myasthenia Gravis Quality of Life scale, it was consistently demonstrated that 75% of patients experienced sustained symptom improvement. These findings were partially confirmed in the phase III study, a randomized, placebo-controlled trial, in which 167 patients with gMG were enrolled and randomized to treatment with either efgartigimod or placebo. The primary endpoint of the study was the proportion of AChR Ab-positive patients with a decrease in the MG-ADL score by ≥2 points after the first 4-week treatment cycle. The results suggested that a significantly higher proportion of patients in the efgartigimod group (44 [68%] of 65) responded to MG-ADL than in the placebo group (19 [30%] of 64), with an odds ratio of 4.95 (95% CI 2.21–11.53, *p* < 0.0001). The MG-ADL response was achieved in 37 (84%) patients by the second week of treatment, suggesting that efgartigimod can lead to rapid and significant clinical improvement in AChR Ab-positive patients. The efficacy was demonstrated by the fact that a high proportion of AChR MG patients in the efgartigimod group (44 [68%] of 65) were cycle 1 MG-ADL responders (primary endpoint). During the study, the efgartigimod group was better tolerated, the incidence of headache was similar between the two groups, and the extent of adverse effects was mild in both groups and not significantly different from the placebo group. Plasma exchange, which is also focused on autoantibody clearance, has a shorter clinical improvement time compared to efgartigimod and requires high operational feasibility and equipment; thus, the FcRn antagonist is expected to become an alternative therapy to PLEX in the future. Furthermore, this class of drugs that can also reduce all IgG concentrations also suggests its potential to be one of the solutions for IgG-mediated autoimmune disorders (Howard et al., 2021). As its rapid action and sustained clinical improvement, FcRn antagonists perhaps exert their function across the severity spectrum of MG, especially in refractory MG (Sivadasan and Bril, 2023). Watanabe K et al. described a female patient diagnosed with anti-acetylcholine receptor antibody-positive myasthenia gravis but who remained in myasthenia gravis crisis even after standard first-line therapies for MG exacerbation, such as plasma exchange, intravenous immunoglobulin, and high-dose corticosteroids, were administered, showing an improvement in MC approximately 12 days after the first application of efgartigimod; in addition, after three cycles, her status changed from refractory MC to minimal symptom expression (MSE), while serial changes in the patient’s serum anti-AChR antibody titer were observed over the course of treatment paralleling the clinical improvement in MG-ADL scale scores following efgartigimod administration. It is important to note that the results in this case may have been influenced by “add-on” effects based on other conventional therapies, and comprehensive data collections involving larger patient population are needed to validate this strategy for MC salvage therapy (Watanabe et al., 2024). Overall, efgartigimod is shown to be well-tolerated and very efficacious, with no widespread immunosuppression; the future development of FcRn antagonists is to be expected.

Another comparable drug, rozanolixizumab, a high-affinity IgG4 monoclonal antibody, has also shown good efficacy. The results of a recently published phase III trial (MycarinG) (Bril et al., 2023) showed that rozanolixizumab significantly outperformed the placebo group in MG-ADL scores at both high and low doses and that patients experienced significant symptom relief. With this endorsement, rozanolixizumab received US FDA marketing approval for the treatment of AChR or MuSK antibody-positive adults with gMG. The trial provides good evidence for the therapeutic direction of neonatal Fc receptor inhibition, and we look forward to obtaining more evidence and application of the efficacy of these drugs in the future.

#### 2.4.4 The efficacy of B-cell activating factor inhibitors

Targeting the B-cell activating factor (BAFF, also known as BlyS), belimumab is a humanized IgG1 monoclonal antibody that prevents BAFF-mediated proliferation and antibody production through its high affinity for the BAFF. Overexpression of BAFF leads to an increase in autoantibodies, and belimumab reduces their levels and maintains B-cell immune homeostasis through its targeting effect (Beecher et al., 2019; Kaegi et al., 2021). In a phase II trial of patients with AChR MG, belimumab neither met the trial primary endpoint of mean change from baseline in the QMG score at week 24 nor did it reflect a difference compared to the placebo group (Hewett et al., 2018). The effect of belimumab in myasthenia gravis patients remains to be confirmed.

Telitacicept (Tai’ai) is a novel recombinant fusion protein that includes the extracellular domain of a transmembrane activator and calcium-modulating cyclophilin ligand interactor (TACI) and a modified human Fc component of IgG (Lee et al., 2021). BLyS, also known as BAFF, is a B-cell activating factor of the tumor necrosis factor (TNF) family, while a proliferation-inducing ligand (April) is associated with the activation of mature B cells and plasma cell antibody secretion (Lee et al., 2021; Xie et al., 2022). Telitacicept can neutralize both BLyS and April, thus effectively blocking the proliferation of B lymphocytes and inhibiting downstream signaling, with more focus on suppression of plasma cells (Shi et al., 2021; Stathopoulos and Dalakas, 2022). As for MG, according to the phase II trial study by Remegen Co., Ltd., patients in the 160-mg dose group showed a mean reduction of 7.7 points in QMG scores, demonstrating significant efficacy (a 3-point reduction in QMG scores is clinically meaningful improvement), indicating that telitacicept can significantly improve the condition of patients with gMG. A phase III trial of telitacicept in myasthenia gravis is currently underway, necessitating more evidences.

#### 2.4.5 The uncertain efficacy of IL-6 antagonists

Cytokines and interleukins, important elements in B-cell pathogenesis, play key roles in promoting inflammation, B-cell differentiation, and subsequent autoantibody production. Tocilizumab is a humanized anti-IL-6 monoclonal antibody that can directly affect the antibody production process. In two refractory MG patients who were unresponsive to rituximab and IVIG, tocilizumab showed good therapeutic efficacy, suggesting that it could be an alternative when rituximab is not effective (Jonsson et al., 2017). However, trial data are too scarce; thus, further exploration of tocilizumab in randomized clinical trials is necessary.

Satralizumab is also a drug that inhibits IL-6 and, thus, reduces autoantibody concentration and has been approved in the treatment of other chronic autoimmune diseases. It has the unique property of dissociating from IL-6 in acidic endosomal pH and entering the circulation of the FcRn pathway to prolong its own half-life and become long-lasting [50.51]. Both tocilizumab and satralizumab are being evaluated in gMG therapy (NCT05067348 and NCT04963270).

## 3 MuSK MG

### 3.1 Clinical feature of MuSK MG

Although most patients with MG have been tested to be positive for AChR Ab, antibodies to muscle-specific kinase are present in a small number of patients. MuSK MG has been reported mainly in adults and rarely in children and the elderly, with prominent symptoms in neck, shoulder, and tongue muscles (Gilhus and Verschuuren, 2015). MuSK MG is predominantly observed in women, with a peak incidence under the age of 40 years (Cao et al., 2020). The first symptom in about one-third of patients with MuSK MG is bulbar muscle weakness associated with respiratory involvement, sometimes with atrophy. When respiratory muscles are involved, myasthenia crisis may occur rapidly and worsen significantly (Marino et al., 2020).

### 3.2 Mechanism of MuSK antibody production

MuSK MG IgG4 autoantibodies are tightly bound to SLPBs. There are two phases in the process of immune memory acquisition, and the final product includes the production of IgG1 by LLPCs, as mentioned above. While in phase 1, the BCR of naive B cells receives Ag presented by the FDC, which, in turn, interacts with Tfh cells and eventually leads to B-cell proliferation. This extrafollicular pathway gives rise to SLPBs, which are cells that mainly produce IgG4, as well as GC-independent memory B cells and GC B cells (Yi et al., 2018; Zografou et al., 2021). The formation of SLPBs expressing non-switched or isotype-switched immunoglobulins can indicate a rapid antigen clearance response (McHeyzer-Williams and Ahmed, 1999). Rituximab-mediated depletion of CD20^+^ B cells, followed by a significant reduction in MuSK autoantibody titers after 3 months, indicates that short-lived antibody-secreting cells (e.g., plasmablasts) are more possible candidates. To date, only the circulation contains MuSK autoantibody-producing B cells, which have memory B cells or short-lived plasmablastic phenotypes (Stathopoulos et al., 2017). Variable proportions of MuSK IgG1, 2, and 3 antibodies were also commonly detected in most patients. The function and value of these antibody subtypes are still controversial (Cao et al., 2020).

### 3.3 Pathogenesis of MuSK MG

**FIGURE 2:**
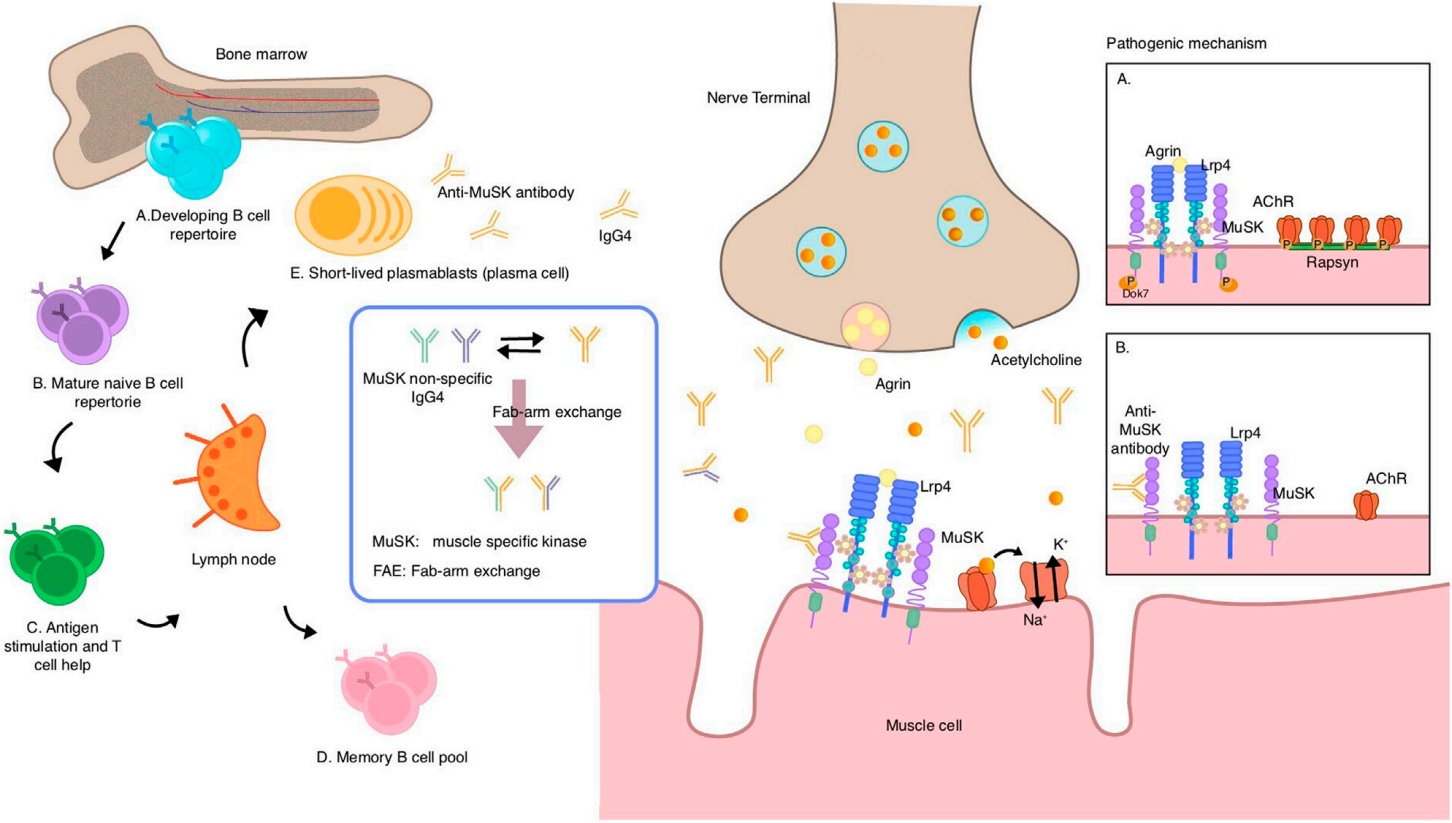
Speculative mechanisms of muscle-specific tyrosine kinase (MuSK) autoantibody production and pathogenic mechanism of MuSK antibodies. MuSK autoantibodies are thought to originate from naive B cells in the bone marrow, which receive T cell help in the lymph nodes and acquire antigenic stimulation, and then differentiate into memory B cells and short-lived plasmablasts that can secrete antibodies. Most MuSK antibodies are of the IgG4 subclass and exhibit the unique ability to undergo fab-arm exchange with non-specific MuSK antibodies, resulting in a transition from a bivalent monospecific form to a monovalent bispecific form known as “fab-arm exchange.” Under normal circumstances, agrin binds to LRP4, thereby activating the agrin/LRP4–MuSK pathway. MuSK then phosphorylates downstream proteins, leading to AChR clustering via rapsyn proteins. **(A)** MuSK antibodies of the IgG4 subclass bind to MuSK, blocking the interaction of MuSK with LRP4, which interrupts the pathway. This prevents AChR from clustering and reduces the density of AChR in the postsynaptic membrane, ultimately impairing neuromuscular transmission **(B)** MuSK muscle-specific tyrosine kinase, LRP4 lipoprotein-receptor-related protein 4.

MuSK is a single-subunit transmembrane protein located on the postsynaptic membrane of the NMJ that plays an important role in clustering the AChR (Gilhus et al., 2019). Normally, agrin, which is synthesized by motor neurons, binds to the MuSK co-receptor LRP4 to trigger the activation of MuSK, thereby clustering the AChR and achieving the maintenance of the postsynaptic membrane. The process of clustering is rapsyn-dependent, which are the proteins that bridge the AChR with the cytoskeleton at the postsynaptic membrane (Conti-Fine et al., 2006). Most of the MuSK Abs are IgG4 (range 63.80%–98.86%), the titer of which corresponds to the severity of the disease and which, due to its unique molecular properties, is very different from the AChR Abs (mainly IgG1 antibodies) in terms of pathogenic mechanisms (Huijbers et al., 2018; Fichtner et al., 2020; Marino et al., 2020). IgG4 can exchange Fab arms with other IgG4 molecules, called Fab-arm exchange (FAE), which means that IgG4 can swap half-molecules with other IgG4 half-molecules (a heavy and light-chain pair). After this process, IgG4 becomes functional, monovalent, and bispecific, that is, it has two Fab arms with different specificities (Huijbers et al., 2018), resulting in its inability to cross-link the AChR (the process requires the binding of two identical fab arms to two antigens). In addition, the shorter hinge region of IgG4 makes it unable to engage in cross-link formation, and the failure of cross-linking in turn prevents endocytosis of the receptors (Fichtner et al., 2020; Zografou et al., 2021). The bispecificity prevents the formation of the antigen–antibody complex and then blocks the immune activation of inflammation; besides, due to its extremely low affinity for C1q complement components, IgG4 cannot trigger the complement cascade via the classical pathway (Fichtner et al., 2020). The absence of these IgG1 properties reflects the immunologically inert and anti-inflammatory properties of IgG4. Experiments have shown that most MuSK autoantibodies are bispecific and have Fab-arm exchange *in vivo*, and *in vitro* measurements of pathogenicity have confirmed that MuSK antibodies do not alter pathogenicity by FAE with IgG4 from healthy sera, implying that monovalent IgG4 is sufficient to induce pathogenicity (Koneczny et al., 2017). The pathogenicity of MuSK autoantibodies is thus thought not to be relied on immune inflammation and complement activation, but exerted through an Fc-independent mechanism also known as the blockade of protein–protein interactions (Koneczny, 2020), where the antibody interferes with the clustering of the AChR by blocking the LRP4–MuSK interaction, thereby disrupting the agrin/MuSK signaling pathway and its maintenance of the integrity of the NMJ structure and function, resulting in impaired neuromuscular transmission and forming symptoms of muscle weakness, the pathogenicity of which has been confirmed by passive transfer studies (Klooster et al., 2012; Gilhus et al., 2019) (Figure 2) Apart from the previously mentioned pathogenic mechanisms, MuSK antibodies possess the subsequent supplementary characteristics: MuSK antibodies that block the involvement of LRP4 in MuSK activation also inhibit ACh vesicle clustering in motor nerve terminals by controlling presynaptic membrane development through the retrograde signal. The pharmacological effects of the symptomatic drug 3,4-diaminopyridine are mediated by increased vesicle release and enhanced conduction (Cao et al., 2020).

Acetylcholinesterase (AChE) binds as tetramers to collagen Q (ColQ), which is anchored to the synapse by an interaction with MuSK. Autoantibodies block the binding of MuSK to ColQ, resulting in the loss of ACh. As an enzyme with the function of hydrolyzing ACh, the reduction in AChE leads to excess ACh accumulation in the synaptic cleft and receptor dispersion, which may explain the hypersensitivity of MuSK MG patients to AChE inhibitors. In studies on nerve conduction, inadequate responses and repetitive compound motor action potentials and fasciculations are evidence of cholinergic neuromuscular hyperactivity in MuSK MG. Moreover, bivalent MuSK antibodies induce MuSK dimerization and activation, which depletes and recruits AChRs, forming ectopic AChR clusters. Given the lack of corresponding motor neuron terminals, ectopic, extra-synaptic AChRs are deprived of neuromuscular transmission (Cao et al., 2020).

### 3.4 Therapeutic strategies for MuSK MG

Patients with MuSK MG are usually unresponsive or intolerant to pyridostigmine or even have cholinergic crisis (Gilhus et al., 2019). IVIG does not work in patients with MuSK MG because of its immunoglobulin nature. Thymectomy has no effect on MuSK MG, and traditional immunosuppressants such as tacrolimus are effective, as is the application of plasma exchange during the acute phase. Plasma exchange, a therapy that takes fresh frozen plasma or replacement fluid such as albumin, is widely used in many autoimmune diseases, with efficacy (Jacob et al., 2021). The elimination of circulating pathogenic antibodies and other humoral factors may explain its ameliorative effect, and efficacy has been observed in neuroautoimmune disorders (Lehmann et al., 2006). Antibodies, complements, cytokines, and other molecules are removed from circulation through plasma exchange (Gilhus et al., 2019). PLEX and IVIG are recommended for short-term, rapid treatment of MG patients with imminent respiratory insufficiency and dysphagia and are not applied for long-term maintenance due to their short duration of action (4–12 weeks) (Narayanaswami et al., 2021). IVIG and PLEX are equally effective and similarly tolerated in patients with moderate-to-severe MG, as demonstrated by Barth et al. (2011). Refractory MG can be periodically performed with PLEX in immunosuppressive therapy-resistant or intolerable patients. As the indication of PLEX, refractory MG is rare, so it is difficult to establish high-value clinical studies to explore it in-depth (Jacob et al., 2021). IVIG exerts a rapid and pronounced effect on AChR MG, yet does not achieve significant improvement in MuSK MG, which is closely related to the unique nature of IgG4. This ineffectiveness can be broadly divided into these mechanisms (Dalakas, 2021): first, IgG1 idiotypic antibodies provided by IVIG cannot neutralize IgG4; second, IVIG can inhibit pathogenic cytokines and immuno-inflammatory molecules and complement binding and MAC formation, while the IgG4 subtype does not possess those properties. In addition, loss of FcyR-related function and competitive degradation also bring futility. Since they are all non-targeted drugs, which can have an impact on immune homeostasis and have high device requirements, the development and application of more specifically targeted drugs is clinically relevant for the comparatively rare MuSK MG.

#### 3.4.1 The efficacy of anti-CD20 therapy

In the past two decades, anti-B-cell therapy targeting important functional molecules on B cells has become a crucial research direction for the treatment of autoimmune diseases (Stathopoulos and Dalakas, 2022). Many novel treatments are emerging, among which rituximab has shown significant efficacy, supporting its use as an option for MG, especially bringing benefits to MuSK MG patients (Gilhus et al., 2019). Rituximab directly targets the CD20 antigen on B cells, causing complement-mediated cytotoxicity that depletes CD20^+^ cells (Cutter et al., 2019; Alhaidar et al., 2022). Mature plasma cells do not express CD20, so short-term humoral immunity is preserved (Marco et al., 2014). Rituximab eliminates CD20^+^ memory and naive B cells but cannot eliminate CD20 plasmablasts or plasma cells, while the significant and rapid decrease in the autoantibody titer indicates that the main antibody-producing cells of MuSK Abs are short-lived plasmablasts rather than plasma cells, reflecting its efficacy of treatment. Notably, MuSK Ab-positive patients treated with rituximab fared better than patients who were positive for other antibodies such as AChR Ab (Cai et al., 2019). International consensus points out that rituximab can be considered an early choice for treatment when initial immunotherapy does not achieve satisfactory results in MuSK MG patients (median 9, range 4–9) because MuSK MG has a higher level of steroid dependence, and earlier studies have demonstrated beneficial effects of rituximab in patients with refractory or severe MG (Alhaidar et al., 2022). According to the prospective review by Hehir et al. (2017), rituximab provides level-IV evidence for treatment of MuSK MG, increasing the likelihood of a positive result. Patients in the rituximab group (58%) achieved a strong and statistically significant clinical benefit compared to the control group (16%), and the efficacy was durable in many patients. A 2017 meta-analysis of 168 patients from case reports and case series assessed the efficacy and safety of rituximab in MG patients with 59% of AChR MG and 34% of MuSK MG. Data analysis showed that rituximab demonstrated efficacy in both MGs, with up to 70% of MuSK MGs reaching minimal clinical manifestation (MM), while only 30% of AChR MGs reached the primary endpoint. Moreover, rituximab showed efficacy, especially in moderate-to-severe refractory MG that had already received several immunotherapy (Tandan et al., 2017). The data provided by Marino et al. (2020) show that rituximab is also safe and can bring long-term benefits to MuSK MG patients. The therapeutic effects of rituximab are mostly due to the decrease in plasma cell precursors, which is supported by consistent findings in other IgG4-mediated disorders. Rituximab had little effect on total IgG4 levels as both total IgG and IgG4 returned to normal levels after several months of treatment. MuSK Ab Antibody Secreting Cells are thought to be short-lived Ab-secreting cells because of the long-term reduction in MuSK Abs, especially IgG4.

Although rituximab has shown favorable efficacy in MuSK MG, there are some minor concerns, such as the relapse of MG. The degree of rituximab induction appears to be directly proportional to the durability of response in patients with MuSK MG (Cortés-Vicente et al., 2018); however, although RTX treatment effectively depletes memory B cells in peripheral blood, some relapses have occurred (Hofmann et al., 2018). It is demonstrated that the precursors to autoantibodies are derived from antigen-experienced CD27^+^ B cells that are affected by rituximab; if the depletion is insufficient, these CD27^+^ B cells can induce the disease to intensify (Lee et al., 2021). Stathopoulos et al. (2017) also suggested that during relapse, CD27^+^ B cells have the specificity of MuSK autoantibody production, and circulating plasmablasts can also spontaneously secrete MuSK-specific antibodies. These results suggest that in a subset of patients, the disease is dormant rather than completely eradicated. Identification of pathogenic clonal variation in MuSK-specific B cells and elevated levels of MuSK autoantibodies prior to relapse both have the potential to be useful prognostic indicators for identifying recurrence following B-cell depletion therapy (Fichtner et al., 2022). In conclusion, more standardized clinical studies on rituximab can provide more evidence for its application and individual regimen in MuSK MG or refractory MG in the future.

Ofatumumab is a fully humanized anti-CD20 monoclonal antibody that is presumed to be better tolerated than rituximab. It has a different binding epitope from rituximab, with a larger range of sites, including not only large loops but also smaller epitopes closer to the B-cell surface, making it more effective with B-cell lysis (Du et al., 2009). Because it has an unmodified Fc region, the affinity of the Fc receptor is increased, and moreover, it also has a C1q-binding site that mediates complement-dependent cytotoxicity (Basu et al., 2022). A patient with refractory MG, who had a poor response to multiple immunotherapies such as rituximab, achieved sustained remission after two infusions of ofatumumab, leading to the speculation that ofatumumab may be one of the treatment options for refractory MG in the future, especially in patients who are poorly tolerant to rituximab (Waters et al., 2019).

Inebilizumab is a monoclonal antibody targeting CD19, which is expressed in a broader range of early pro-B cells, plasma cells, and plasmablasts compared to CD20. Inebilizumab achieves its pharmacological effects through antibody-dependent cell-mediated cytotoxicity of depleting B cells (Nair and Jacob, 2023). The drug is being evaluated in an ongoing phase III clinical trial (MINT) enrolling patients with generalized AChR MG and MuSK MG, and its efficacy and safety remain to be investigated (Dalakas, 2020).

#### 3.4.2 Ineffectiveness of eculizumab in MuSK MG patients

The anti-complement agent eculizumab has very little therapeutic effect on MuSK MG patients, which is closely linked to its pathological mechanism. The complement is a circulating protein that complements antibodies and phagocytes, thereby inducing a cascade response, and the complement response is a potent inflammatory process (Lee et al., 2021). Complement-induced damage to the postsynaptic membrane is one of the causes of the symptoms of myasthenia gravis (Gilhus et al., 2019). By inhibiting terminal complement activation, eculizumab particularly interacts with complement protein C5, thereby diminishing membrane destruction. Its potential benefit for application in AChR Ab-positive refractory generalized MG has been confirmed by the phase III REGAIN study (Howard et al., 2017). However, IgG4 functionally differs from other IgG subtypes and does not induce activation of the complement response due to its low affinity for C1q, which is the q fragment of the first element of the complement (van der Neut Kolfschoten et al., 2007). Moreover, the complement-inducing IgG1 subtype, which is the main type of AChR MG, is the effective target of complement inhibitors; therefore, eculizumab and virtually all drugs in this category have nothing to do with the pathogenic mechanism of IgG4, which is the main pathogenic antibody type of MuSK MG, by virtue of their pharmacological action and, thus, may not be effective.

#### 3.4.3 Ineffectiveness of FcRn antagonists in MuSK MG

The representative FcRn antagonist efgartigimod, which reduces the pathogenic antibody concentration by decreasing the half-life of IgG, has shown efficacy and safety in both phase II and phase III trials of patients with gMG. However, the effect of efgartigimod on MuSK MG or on IgG4 still needs to be confirmed in further trials. It is currently known that a rapid and significant reduction in levels of all IgG and IgG subtypes could be observed in the phase II trial conducted by Howard et al. (2021). However, in the phase III ADAPT trial (Howard et al., 2019), although patients with MuSK antibody-positive and double-negative MG have been included, the effectiveness of the drug against these subtypes could still not be demonstrated. Although FcRn inhibitors were able to reduce circulating levels of all IgG subtypes, they have not been formally tested in MuSK MG patients (Fichtner et al., 2020). In another study conducted by Ulrichts et al. (2018) exploring intravenous single ascending dose (SAD) and multiple ascending doses (MADs) of efgartigimod, both SAD and MADs for each IgG subtype remained interestingly similar after dosing, and IgG2 and IgG3 decreased over time similarly to IgG1, but IgG4 seemed to have a slightly smaller reduction. This may be related to the different production rate of IgG4 and the different mode of interaction with FcRn, although there is no trial to provide evidence. However, the results of a new study aimed at exploring the efficacy of the FcRn antagonist rozanolixizumab (MycarinG study) suggest that the drug has efficacy in not only AChR but also MuSK gMG patients. The same FcRn inhibitors such as batoclimab that the result of phase III trial may be available next year (Yan et al., 2022). In the future, FcRn antagonists may provide benefit to patients with different types of antibodies (Bril et al., 2023). In conclusion, it is undetermined whether FcRn antagonists are feasible in the treatment of patients with MuSK MG, and more clarification of relevant trials is expected.

#### 3.4.4 Efficacy of BAFF inhibitors

**FIGURE 3:**
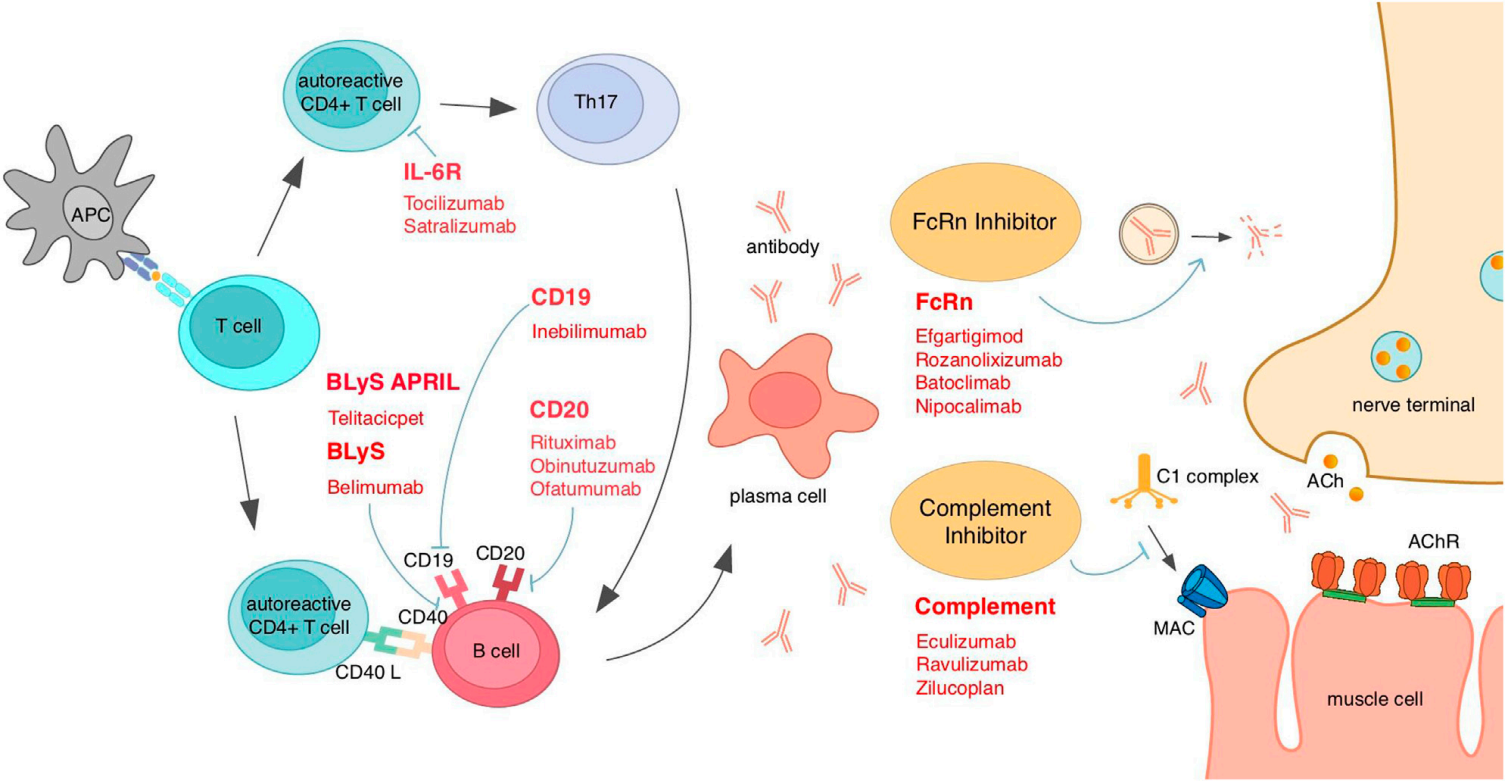
Existing emerging biologics and interventions based on MG mechanisms for different targets. *AChR*, acetylcholine receptor; *APC*, antigen-presenting cells; *April*, a proliferation-inducing ligand; *BLyS*, B-lymphocyte stimulator; *FcRn*, neonatal Fc receptor; *IL*, interleukin; *MAC*, membrane attack complex.

Telitacicept, as a biological agent with dual targets of BLyS and April, can bind and neutralize those two target molecules and, therefore, disrupt B-cell homeostasis and inhibit B-cell development (Dhillon, 2021). B cells have the function of secreting autoantibodies, which is vital for MG pathology; thus, targeting B cells is a common direction for the treatment of AChR MG or MuSK MG. The future application of this class of drugs like telitacicept in MuSK MG patients is also expected, and more trials are needed (Figure 3).

## 4 Seronegative MG

Myasthenia gravis can be divided according to the type of autoantibodies into AChR antibody-positive MG, MuSK antibody-positive MG, and a small percentage of MG with no detectable antibodies to either of these, which is called double-negative MG (DNMG). In a subset of these patients, anti-LRP4 antibodies can be detected as LRP4-positive MG (LAPMG). MG that is not detected by any of the above three antibodies is defined as seronegative MG, which accounts for about 10% of patients with generalized MG (Gilhus et al., 2016). An acceptable definition of seronegative MG is a weak or fatigued patient with electrophysiological evidence of impaired neuromuscular junction conduction, but no abnormal autoantibodies against neuromuscular junction components are detected in routinely examined serum. The term “seronegative MG” is unfavorable because of its ambiguity since, with the improvement of detection techniques, both MuSK and LRP4 antibodies have been identified from patients once defined as seronegative MG by studying new targets (Fichtner et al., 2020).

### 4.1 Clinical features of seronegative MG

The clinical features of double-negative MG cannot be generalized because the causative mechanisms are not clear, and this category is heterogeneous. However, there are some common features that can be summarized. Some studies report female predominance. Bulbar muscle involvement and impaired respiratory function occur less frequently, and symptoms present as milder than in antibody-positive MG (Argov, 2011). In triple-seronegative myasthenia gravis that is anti-AChR-MuSK-LRP4 antibody-negative, ocular muscle involvement (33%) manifested more significantly than AChR MG (13%) (*p* = 0.0250) (Morena et al., 2022). Since its course is usually mild, it adds to the diagnostic challenge. In addition, although very rare, this study suggests that investigation of thymic pathology should be considered in SNMG. In a multicenter study of anti-LRP4/agrin antibody-positive MG, it was revealed that 15% of DNMG is LAPMG and that LAPMG has more severe symptoms and a longer duration of disease (Rivner et al., 2020). However, the detection rate of positive LRP4 antibodies varies widely by the test method and population. The majority of patients with either DNMG or LAPMG responded to standard treatment, suggesting that they are both immune diseases with autoimmune pathology. This study also found that most patients with antibodies against LRP4 also had antibodies against agrin, which binds to LRP4 and other proteins on muscle cell membranes and controls the development, maintenance, and regeneration of neuromuscular junctions. Antibodies against agrin and LRP4, which are essential extracellular functional molecules in the MuSK signaling pathway, are therefore highly suspected to be associated with disease pathology, although their pathogenicities remain unknown (Gilhus et al., 2019). Given the severe and long-lasting nature of LAPMG, the detection of LRP4/agrin antibodies in DNMG and, thus, the prediction and prognosis of disease are of great interest for the development of treatment strategies (Rivner et al., 2020). Another multicenter analysis was performed on seronegative myasthenia crisis (SNMC), a life-threatening critical condition of myasthenia gravis without antibodies to either AChR or MuSK (Mergenthaler et al., 2022). Compared with AChR MG crisis, SNMC had a younger age of onset (54.3 ± 14.5 vs. 66.5 ± 16.3 years; *p* = 0.0037), a longer time between the onset of MG and the crisis (8.2 ± 7.6 vs. 3.1 ± 4.4 years; *p* < 0.0001), a higher incidence in female patients, and a greater tendency to have thymic hyperplasia. The possibility that some of the SNMG patients may have thymic hyperplasia is also reflected in the report by Leite et al. (2008). The thymus has the function of producing autoantibodies in MG pathology, so it is reasonable to suspect that patients with seronegative pathology may have low-affinity antibodies, but other hypotheses cannot be ruled out due to its heterogeneity (Mergenthaler et al., 2022). A study of drug-refractory MG found that 80% of seronegative drug-refractory MG does not respond to any drugs, which means that seronegative patients are more probable to be drug-refractory and less likely to be treated with improvement, so an investigation of the mechanism and development of new drugs is necessary and urgent since drug-refractory conditions are very serious (Cortés-Vicente et al., 2022).

### 4.2 Mechanistic hypotheses of seronegative MG

There are many speculations as to the cause of negative serum antibody test results in MG patients, some of which were somehow confirmed in clinical trials, but the evidence is not sufficient and reliable. As early as in 2003, the reason for seronegative MG may be the conjecture that the low affinity of the antibody leads to them being undetectable by conventional methods or activation of the second messenger pathway by non-IgG plasma factor (Vincent et al., 2003). With the development of technology, more and more new detection techniques have emerged, bringing innovation to the detection of seronegative MG. Jacob et al. (2012) used cell-based assay (CBA) to identify clustered AChR Abs in approximately 60% of patients with previously seronegative MG, and a proportion of these antibodies was also present in seronegative ocular MG patients. The clustered AChR MG ab is included in the range of low-affinity antibodies that were initially assumed seronegative and belongs to the IgG1 class, which has complement-activating properties. The deposition of the complement is associated with increased jitter in single-fiber electromyography (SFEMG), suggesting that these antibodies are pathology related since electrophysiological defects can be observed (Jacob et al., 2012). Hong et al. (2017) tested patients previously diagnosed as seronegative by a sensitive and novel method, and one-third of them presented positive for at least one antibody, hence reflecting the need for innovation in detection tools. As for the hypothesis of a certain plasma factor, Plested et al. (2002) mentioned that a plasma factor capable of inhibiting AChR function was confirmed in a proportion of SNMG in the test, which binds to non-AChR receptors and activates the second messenger pathway, eventually leading to AChR function defects, but its inhibitory effect is transient. This inhibitory molecule is highly suspected to be of the IgM class, but its nature cannot be determined due to the unknown target antigen. This test provides the basis for the conjecture above.

T-cell immunity without antibodies highlights the significance of AChR-specific T cells in MG (Karni et al., 1997). Lymphocytes from patients with SNMG are at least partially sensitive to AChR, given that peripheral blood lymphocytes from SNMG patients can respond to peptides of the AChR subunit sequence. T cells, as lymphocytes involved in the pathogenesis of MG, show their importance in MG through the binding of antigen-presenting cells (APCs) to these peptides. Guptill et al. (2021) showed in an exploratory study CD8^+^ IFNγ-producing T cells in borderline association with MG. There is evidence that the lesser noticed CD8^+^ T-cell population and IFNγ with strong cytotoxicity frequency in AChR MG have increased, which raises concern for the investigation of CD8^+^ T cells in myasthenia gravis, including SNMG. Immune checkpoint inhibitors (ICIs) are drugs that target T-cell surface inhibitory molecules and stimulate immune upshift to achieve anticancer effects. Immune checkpoints include cytotoxic T-lymphocyte-associated protein 4 (CTLA-4) and programmed cell death protein 1 (PD-1) (Dubey et al., 2020). Its mechanism leads to a plethora of multisystem immune-related adverse reactions, including neurodisorders like MG (Narayanaswami et al., 2021). In a review, 1,834 patients were treated with ICIs, four had MG, and one of them was AChR Ab-positive (Lazaridis and Tzartos, 2020). Seronegative MG is more common in such patients, suggesting a possible effect of T-cell immunity in its pathogenesis and making the corresponding treatment challenging.

**FIGURE 4:**
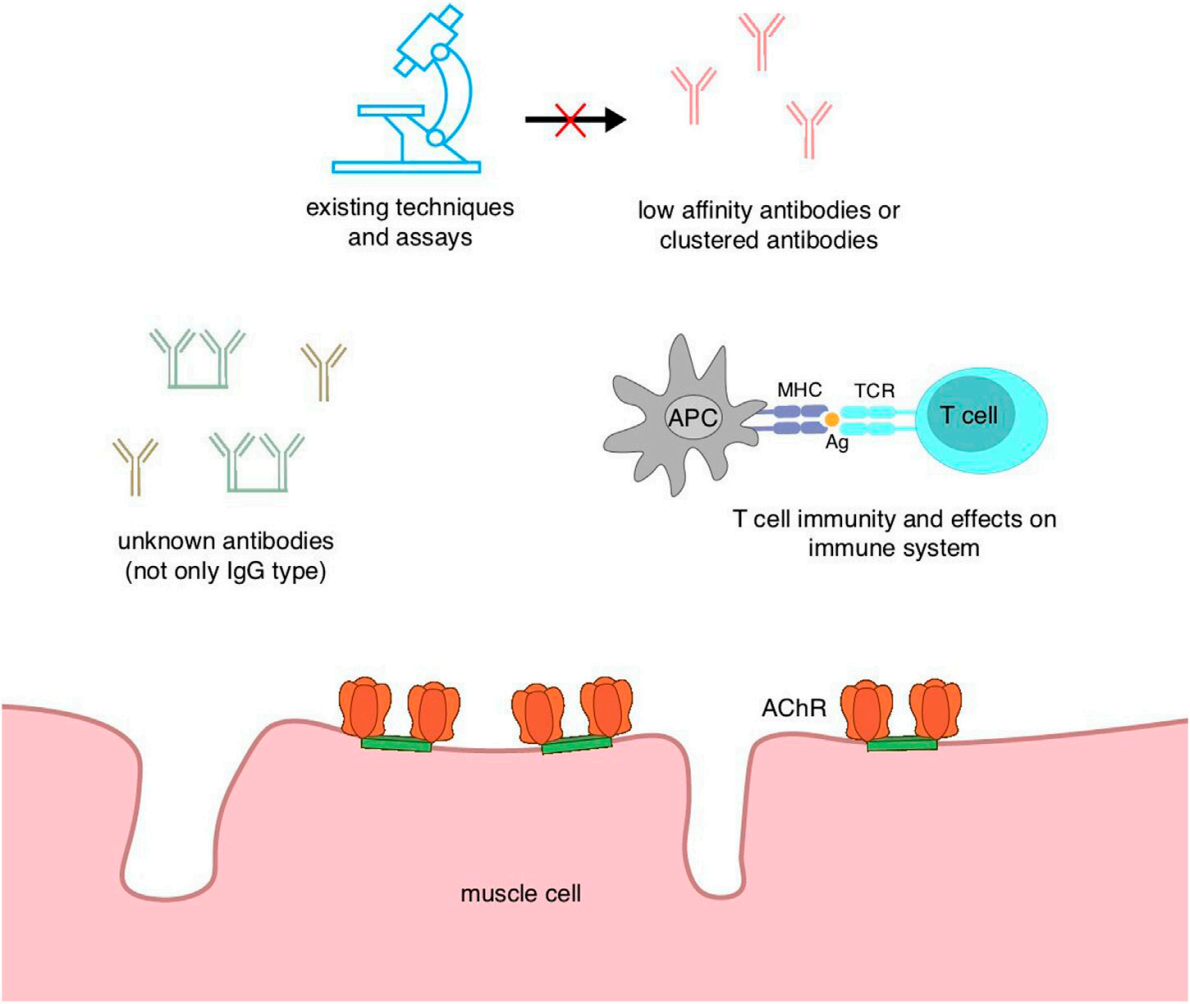
Diagram of hypotheses of seronegative myasthenia gravis.

Patients currently diagnosed with seronegative or double-negative myasthenia gravis subgroups may be due to insufficient levels of existing conventional assays or may have antibodies against unknown targets. Therefore, it will be beneficial to improve the sensitivity of the assays and even develop new and more effective methods of detecting antibodies for disease grouping and clinical guidance of treatment. CBA is able to detect AChR antibodies that cannot be measured by other methods, although its accuracy is not as good as that of radioimmunoprecipitation assay (RIPA; the current gold standard for autoantibody detection) (Lazaridis and Tzartos, 2020), and IgG-specific MuSK CBA has been reported to detect potential MuSK antibodies in SNMG patients and has been suggested to be tested alongside clustered AChR and LRP4 CBA for SNMG (Huda et al., 2017). A sensitive method for detecting titin antibodies has also been developed. Traditional enzyme-linked immunosorbent assay (ELISA) can only detect titin antibodies in AChR MG patients (Hong et al., 2017). Titin is a filamentous muscle protein in striated muscle cells, and despite the huge size, titin antibodies usually only bind to MGT30, a specific 30-kDa segment (Lazaridis and Tzartos, 2020). The presence of titin antibodies has an age-related profile, with a higher prevalence in late-onset MG, and in early-onset MG, it is associated with a high frequency of thymoma. The presence of titin antibodies is suggestive of a more severe disease condition. Usually, titin antibodies are measured only in AChR MG, but Stergiou et al. (2016) detected titin antibodies in seronegative MG patients. The pathogenesis of the disease may be due to as yet undetected antibodies and the existence of titin antibodies due to bystander effects. Nonetheless, titin antibodies may still serve as a biomarker for SNMG, providing a certain degree of diagnostic assistance. Another suspected antigen of double-negative MG, cortacin, has also been shown to be relevant in trials. Cortacin is a protein that acts downstream of the agrin/LRP4/MuSK signaling pathway and is associated with clustering of AChR. Double-negative MG possessing antibodies against cortacin exhibits milder generalized and ocular MG, and the detection of cortacin antibodies of seronegative MG predicts the diagnosis of ocular MG (Cortés-Vicente et al., 2016). However, cortacin antibodies are also found in other immune diseases, so the specificity is not good, and its predictive value needs to be evaluated before practical clinical application (Lazaridis and Tzartos, 2020). In addition, there are some extracellular and intracellular antibodies of interest, such as extracellular Kv1.4 antibodies, ColQ antibodies, and intracellular rapsyn antibodies, but their diagnostic value cannot be determined due to their lack of clinical association or low specificity, but they are important to open up horizons for SNMG that cannot be detected by existing assays. Moreover, they serve as biomarkers to provide predictive and diagnostic aids for disease and to even subsequently explore unknown autoantibodies, which are worthy research directions (Figure 4).

The diagnosis of seronegative MG as “seronegative” may be due to the lack of specificity of the assays and the fact that the current state of the technology does not enable the detection of antibodies. Furthermore, the low affinity of the antibodies and the variable concentrations hinder their detection. Second, there may still be some as yet unidentified pathogenic antibodies, and these unknown antibodies need further investigation. Furthermore, T-cell immunity may be associated with seronegative MG; however, the available evidence is insufficient, and a deeper understanding of the immune mechanism is necessary.

### 4.3 Treatment outlook of seronegative MG

Confirmation of the diagnosis and treatment strategy for double-negative MG and even seronegative MG is complex and challenging. With repeated autoantibody testing over time, diagnostic corrections may occur due to increased antibody concentrations and increased sensitivity of the assay. Thus, patients should not be defined as “seronegative” directly, but should undergo different measurements over time to rule out other possibilities. Retesting after 6–18 months in patients with an initial diagnosis of SNMG is recommended (Gilhus et al., 2016). Although SNMG shows a tendency to respond well to conventional therapy, similar to the therapeutic responsiveness of the AChR, i.e., good response to acetylcholinesterase inhibitors and positive response to immunosuppressive therapy (Leite et al., 2008), achieving an accurate diagnosis of seronegative MG is still necessary to make individualized treatment judgments. Repeatable and efficient assays are needed, but the cost of novel technologies and their accessibility in different levels of hospitals pose limitations to accurate diagnosis. Guptill et al. (2021) concluded that an increase in CD19^+^CD20^−^CD38^hi^ plasmablast frequencies was associated with a lower likelihood of SNMG, which means that a decrease in plasmablast frequencies was strongly associated with the diagnosis of SNMG. This brings a whole new vision for the search and identification of biomarkers for the diagnosis of MG. Immune cells like plasmablasts that are involved in immunopathological processes have the potential to become biomarkers, providing hope for the future discovery of biomarkers with the potential to evaluate treatments and simplify clinical trials. Given that SNMG diagnosis is chronically time-consuming, and insurance claims are not met due to the uncertainty of the diagnosis, thus imposing a disproportionate financial and psychological burden on patients, the exploration and application of new technologies and new biomarkers and the adaptation of existing therapies tailored to each patient are spots that deserve attention and research.

## 5 Other intervention therapies

Thymic overexpression of interferon (IFN)-β and IFN-I induced genes is observed in MG patients. IFN-I, and especially IFN-β, appears to be the orchestrator of the thymic changes. Therefore, anti-IFN therapy may be a new direction for targeted therapy in MG. Furthermore, through the degradation of messenger RNAs, which stops translation and the synthesis of proteins, miRNAs can control the expression of genes that are post-transcriptionally expressed. The Experimental Autoimmune Myasthenia Gravis mouse model suggests that a decrease in miR-29a/b1 could promote the upregulation of IFN-β and the formation of pro-inflammatory Th17 cells, potentially influencing MG susceptibility. Future MG treatment options may be inspired by monitoring or even modifying miRNAs (Cron et al., 2020). The effects of chimeric antigen receptor (CAR) T-cell therapy, one of the standard treatments for cancer, are being investigated in autoimmune diseases. Genetically altered T cells express CARs, which are directed against B cells that secrete autoantibodies (Menon and Bril, 2022).

## 6 Conclusion

Early conventional immunosuppressive drugs were effective, but a series of adverse events brought great physical and psychological burdens to patients, especially those who have been using them for a long-term period. In recent years, it is not only myasthenia gravis but also many neuroimmune diseases that have gone beyond the conventional agents to a new era of targeted biologics development, with new drugs being developed in an endless stream and targeting diverse sites, creating new possibilities for the treatment of myasthenia gravis, an autoimmune disease. In the face of a vast array of new drugs, how to choose and apply them in order to achieve a targeted approach and maximize their effects is a direction worth exploring and of clinical value. Accurately identifying and subgrouping autoantibodies in patients plays a huge role in the subsequent tailoring of therapeutic strategies and prognosis according to the characteristics of different antibodies. Novel drugs that are effective against AChR MG include complement inhibitors such as eculizumab, FcRn antagonists such as efgartigimod, and BAFF inhibitors such as belimumab and telitacicept. As for MuSK MG, anti-CD20 agents have proven to be efficient, such as rituximab, and likewise, BAFF inhibitors (Table 2). The response of seronegative MG to conventional therapies is similar to that of AChR in broad terms, but the difficulty of making a definitive diagnosis and the rarer incidence of the disease create difficulties in the application of the drugs, especially with the newer drugs on the market and the dose adjustment of the drugs already applied. The timing of drug initiation deserves consideration, and a trial of rituximab in new-onset and refractory non-MuSK MG concluded that the initiation of rituximab at an early stage can be beneficial, suggesting that early initiation of the targeted drug is currently preferable (Brauner et al., 2020). However, there are risks associated with switching drugs in patients who have already had good prior treatment and are in a stable state, which need to be considered with discretion. It is also worth noting whether the tapering of conventional drugs after drug switching in refractory MG results in fewer adverse effects and better tolerance. Each aspect of how to switch from conventional to novel medications, from combination to even complete substitution, requires a large body of evidence to provide data support, and there is currently a relative void in the field. Yet, for conditions that are common in patients with MG, those with other comorbidities in the past, the combination of immunologic drugs is recommended. Despite the hope offered by targeted medications, there are many trivial issues that should not be neglected as they determine whether the medication will be successfully utilized and helpful to the patients. The way the drug is administered, i.e., oral, intravenous, or subcutaneous, the frequency of administration, and the cost of the drug all affect the willingness of patients to choose the drug and medical compliance. The high financial burden and uninsured status of new medications limit many patients’ access to them and may even delay remission. The selection of drugs for different populations requires careful assessment and trial basis; children, pregnant women, and the elderly are usually not included in the inclusion criteria of clinical trials due to higher risk. Diagnosis and treatment are necessary, but there is a lack of evidence to support the use of medication, which demands a more precise and confident design and investigation of trial protocols. Children with generalized myasthenia gravis administrated the FcRn antagonist efgartigimod are being recruited for a clinical trial (NCT04833894) and can be followed consistently in the future. The emerging phase II or phase III clinical trials have demonstrated the effectiveness of targeted drugs, but generally, the trial duration is short and should take into account the relapsing-remitting nature of MG and the insufficiency of the long-term profile of efficacy and safety, and the need for high-quality randomized controlled trials has never ceased. The improvement of novel antibody assays has helped us classify patients more accurately for diagnosis, giving support to the development of advanced targeted drugs and making target-specific individualized immunotherapies with fewer side effects and faster onset of action more common. Optimizing existing tools to ultimately improve the quality of life of patients, improve disease management, and achieve the ideal state of living with the disease but no aggravation is a vision shared and expected by all clinicians and patients.
